# Challenges, opportunities and solutions for local physical activity stakeholders: an implementation case study from a cross-sectoral physical activity network in Northeast England

**DOI:** 10.1186/s12889-020-09847-3

**Published:** 2020-11-23

**Authors:** Benjamin P. Rigby, Peter van der Graaf, Liane B. Azevedo, Louise Hayes, Benjamin Gardner, Caroline J. Dodd-Reynolds

**Affiliations:** 1 Department of Sociology, Faculty of Social Sciences and Health, Durham University, 32 Old Elvet, Durham, DH1 3HN UK; 2 NINE Doctoral Training Partnership, C/O Faculty of Social Sciences and Health, Durham University, Arthur Holmes Building, Durham, DH1 3LE UK; 3 Wolfson Research Institute for Health and Wellbeing Physical Activity Special Interest Group, Durham University Queen’s Campus, University Boulevard, Thornaby, Stockton-on-Tees, TS17 6BH UK; 4 Fuse: The Centre for Translational Research in Public Health, Newcastle University, Newcastle-upon Tyne, NE1 7RY UK; 5 Centre for Public Health Research, School of Health and Life Sciences, Teesside University, Southfield Road, Middlesbrough, TS1 3BX UK; 6 Department of Allied Health Professions, Sport and Exercise, School of Human and Health Sciences, University of Huddersfield, Queensgate, Huddersfield, HD1 3DH UK; 7 Population Health Sciences Institute, Newcastle University, Baddiley-Clark Building, Richardson, Newcastle, NE2 4AX UK; 8 Institute of Psychiatry, Psychology and Neuroscience, King’s College London, Denmark Hill, London, SE5 8AF UK; 9 Department of Sport and Exercise Sciences, Durham University, 42 Old Elvet, Durham, DH1 3HN UK

**Keywords:** Physical activity, Translational research, Policy, Networks, Complexity

## Abstract

**Background:**

Increasingly, national policy initiatives and programmes have been developed to increase physical activity (PA). However, challenges in implementing and translating these policies into effective local-level programmes have persisted, and change in population PA levels has been small. This may be due to insufficient attention given to the implementation context, and the limited interactions between local policy-makers, practitioners and researchers. In this paper we use a case study of a cross-sectoral network in Northeast England, to identify the local-level challenges and opportunities for implementing PA policies and programmes, particularly the updated 2019 UK PA guidelines.

**Methods:**

Five focus groups (*n* = 59) were conducted with practice partners, local policy-makers and researchers during an initial workshop in April 2018. Through facilitated discussion, participants considered regional priorities for research and practice, along with barriers to implementing this agenda and how these may be overcome. During a second workshop in December 2018, overarching findings from workshop one were fedback to a similar group of stakeholders, along with national policy-makers, to stimulate feedback from delegates on experiences that may support the implementation of the UK PA guidelines locally, focusing on specific considerations for research, evidence and knowledge exchange.

**Results:**

In workshop one, three overarching themes were developed to capture local challenges and needs: (i) *understanding complexity and context*; (ii) *addressing the knowledge and skills gap*; and (iii) *mismatched timescales and practices*. In workshop two, participants’ implementation plans encompassed: (i) *exploring a systems approach to implementation;* (ii) *adapting policy to context*; and (iii) *local prioritising.*

**Conclusions:**

Our findings suggest that academics, practitioners and policy-makers understand the complexities of implementing PA strategies, and the challenges of knowledge exchange. The updated UK PA guidelines policy presented an opportunity for multiple agencies to consider context-specific implementation and address enduring tensions between stakeholders. An organically derived implementation plan that prioritises PA, maps links to relevant local policies and supports a context-appropriate communication strategy, within local policy, practice and research networks, will help address these. We present 10 guiding principles to support transferable knowledge exchange activities within networks to facilitate implementation of national PA policy in local contexts.

**Supplementary Information:**

The online version contains supplementary material available at 10.1186/s12889-020-09847-3.

## Background

Physical inactivity is a global health issue and a leading risk factor for non-communicable disease and mortality [1]. Global estimates indicate that 28% of adults and 81% of adolescents do not meet PA recommendations for health [2, 3]. In the United Kingdom, the figures are 36.7% for adults and 53.2% for children and young people [4, 5]. Furthermore, inequalities in PA participation persist within and between countries, in relation to many socio-demographic factors such as gender, socio-economic status and the built environment [1, 6, 7].

Persistent global physical inactivity has resulted in a heightened policy response at international and national levels [1, 8]. The recent *Global Action Plan on Physical Activity* set out a framework to guide national-level decisions, and presented a suite of effective and feasible policy actions for countries to promote PA, according to their specific requirements [1]. While national PA plans and campaigns are increasingly common in both high and middle income countries [8–10], the UK’s most consistent policy response has been the publication of robust, evidence-based recommendations [11]. Their purpose is to provide key stakeholders with guidelines on the volume, duration, frequency and type of PA required across the life-course to achieve health benefits; this was reinforced in the updated *UK Chief Medical Officers’ Physical Activity Guidelines,* published in September 2019 [12].

*Everybody Active, Every Day* [13] was published to support the implementation of previous guidelines and present an evidence-based plan for PA in England across four domains (i.e. active society, moving professionals, moving at scale, and active environments). While this coherent policy successfully integrated several other policy areas, it may need to be updated in light of the UK’s new guidelines, and remains just one of several national-level policies (e.g. the *Childhood Obesity Plan* [14], the *Cycling and Walking Plan* [15], or the *Sporting Future* strategy [16]. Combined with piecemeal, and sometimes contradictory, public education campaigns, target setting and surveillance [11], there is arguably no overarching national UK strategy per se. These factors have contributed to confusion about how active people should be and how best to implement policies to support population behaviour.

Moreover, national-level approaches have typically overlooked the complexities of population behaviour change [17]. The UK policy sphere has emphasised individual lifestyle choices [18, 19], thus detracting from the wicked nature of physical inactivity (i.e. a socio-cultural issue that is difficult to solve completely). This emphasis has been bound-up in discourses that characterise physical activity as a health-enhancing product that people should consume, for the betterment of themselves and as a moral contribution to society [19]. This individualistic approach fails to recognise that increasingly, diverse technical expertise is required to consider the multiple interacting causes of inactivity, and the range of political interests and actors involved is extending further beyond traditional health sectors (e.g. to environment, education, transport and business) [20, 21]. Combined, these issues make the development and implementation of programmes particularly challenging. To enhance implementation, it is important for policy to recognise that mechanisms underpinning effective PA programmes will differ by person, context and place [22, 23]. For example, compared to men and younger adults, women and older adults more readily engage in exercise referral schemes and outdoor walking groups [24, 25].

In this inherently complex context, we argue that national policies are necessary but not sufficient to address physical inactivity, without significant local-level stakeholder involvement and attention to local needs, which is often overlooked. Evidence indicates that additional bottom-up insights and interests developed by, or representing experiences of, those directly involved in policy delivery at a local ground level may generate detailed accounts of policy implementation and support PA in specific populations and contexts [26, 27]. Such insights are important in implementing national policy, as well as developing local policy and community initiatives. However, difficulties arise when transferring bottom-up approaches to new contexts. Policy and implementation frameworks created from them are often based on limited information and complex heuristic models of the implementation context [28, 29].

These implementation challenges have been highlighted in previous studies that emphasise the need for sustained collaboration between practice, policy and research at different system levels. For example, a supportive interface may overcome incompatible individual behaviours of policy-makers, practitioners and researchers, if it is coherent across system levels, including from the top-down (i.e. those at higher-levels that are more removed from ground-level experiences) and the bottom-up [30]. Furthermore, it is important to harness vertical communication (between the actors of system levels) and horizontal communication (between the actors of policy, practice and research at each system level) [30]. Particularly, this latter element is often overlooked in developing national policy [31].

We propose that a convergence of top-down and bottom-up approaches is required to change the culture around PA, and increase the likelihood of success in programmes to promote PA engagement [17, 32]. To achieve this, it is important to explore what the role of stakeholders in implementing PA policy is locally, and how this can be optimised. This may help stakeholders identify and address conflict that can arise between key stakeholder groups, for example due to their relationships and aspirations, in the delivery of grassroots initiatives in implementing ambiguous national PA policy [17].

### A case study of local convergence between policy, practice and research: Fuse Physical Activity Network

In this paper, we present activities undertaken by a regional Physical Activity Network (PAN), hosted by Fuse, the Centre for Translational Research in Public Health [33], as a case study to identify conditions that may support the implementation of national policy at a local level.

Fuse was established and funded by the UK Clinical Research Centres (UKCRC) in 2008 and is one of five Public Health Research Centres of Excellence. To ensure evidence-informed practice through the pooling of expertise and the co-creation and translation of high-quality relevant and useable research, Fuse’s innovative and collaborative approach to translational research has been to use local stakeholder and public engagement, to build relationships and influence the development of regional agenda for policy, practice and research. This work includes a fully developed communications function and knowledge brokerage.

Over the last 10 years, Fuse has developed a practical model for knowledge exchange [34] to support the use of research evidence to inform policy and practice. Much of the current knowledge exchange literature is concerned with conceptual models and frameworks [35–38], and there is a lack of empirical studies that illustrate how to apply these models in practice [39, 40]. The Fuse model provides a practical understanding of how research evidence can be localised and tailored to address translational barriers [41] and puts a strong emphasis on the relational dimension of these activities [42]. The model proposes four steps in the knowledge exchange process: 1) awareness raising; 2) sharing knowledge; 3) making evidence fit for purpose; and 4) supporting uptake and implementation of evidence.

To support knowledge sharing about PA between practitioners and researchers in the region (step 2), Fuse hosts an active PA research network (Fuse PAN). This network enables the two-way communication of views, the sharing of different knowledge types and joint activity. Fuse PAN workshops provide opportunities for networking and the dissemination of research and evidence gathered from across sector boundaries.^1^ They promote opportunities for dialogue and knowledge exchange which helps to facilitate new research and support implementation of PA strategies.

The research network has developed organically over the years through a perceived social responsibility to challenge research boundaries, to embrace innovation from Northeast practice partners, and invite new methods and approaches that contest the PA status quo. At the time of writing, the Fuse PAN was led by a small team of regional academics (*n* = 4), a senior practice partner (*n* = 1) and postgraduate research students (*n* = 2). It has provided a platform to discuss distinct PA themes, varying from PA measurement to inequalities, and enabled these topics to be explored in the context of the needs and perspectives of local stakeholders.

In this paper, we use the Fuse PAN case study to focus on the interplay of different PA stakeholders within the wider PA system, including regional and national policy-makers to:

1. Explore local challenges and opportunities for researchers and practitioners to collaborate on research, policy development and implementation.
2. Explore how local and national stakeholders can work together to implement the updated national UK PA guidelines.
3. Develop a set of guiding principles that will foster future research and support the design and implementation of PA policies and interventions.

## Methods

Data were collected in two regional Fuse PAN workshops.

### Workshop 1: 20th April 2018 (Newcastle, UK)

#### Keynote discussions

During the first workshop held 20th April 2018 (Newcastle, UK), stakeholders from academia (e.g. lecturers and students), policy (e.g. civil service and local authority public health) and practice (e.g. sport development and health improvement) discussed three topics from previous meetings that a survey of PAN members identified as continued local priorities: i) PA, children and young people; ii) workplace PA; iii) PA and health inequalities. After keynotes introducing each topic, delegates were invited to take part in focus groups [43].

#### Focus groups: selection and participants

Delegates self-selected a focus group topic based on the keynote presentations, to reflect their interests and expertise. All five focus groups were simultaneously conducted during the afternoon: two focused on ‘PA, children and young people’ (C1 and C2), one focused on ‘workplace PA’ (W1), and two focused on ‘PA and health inequalities’ (I1 and I2). Table 1 provides the number of participants in each focus group according to sector. The authors were also facilitators or participants and are included in the sample below.

**Table 1:** Focus group participants (n = 59) by employment sector and topic

Participants’ employment sector	Focus group topics			
	PA, children & young people	Workplace PA	PA & health inequalities	Total
**Higher education**	**12**	**6**	**14**	**32**
*Academic*	*6*	*6*	*6*	*18*
*Doctoral studies*	*6*	*–*	*6*	*12*
*Professional support*	*–*	*–*	*2*	*2*
**Government**	**4**	**7**	**7**	**18**
*Local authorities*	*4*	*7*	*3*	*14*
*Non-departmental public bodies*	*–*	*–*	*4*	*4*
**Voluntary & commercial**	**2**	**1**	**4**	**7**
*Sport and PA development*	*2*	*–*	*3*	*5*
*Exercise referral*	*–*	*–*	*1*	*1*
*Trade unions*	*–*	*1*	*–*	*1*
**Primary care**	**–**	**1**	**1**	**2**
*Exercise therapy*	*–*	*–*	*1*	*1*
*Physiotherapy*	*–*	*1*	*–*	*1*
**Totals**	**18**	**15**	**26**	**59**

#### Focus groups: methodology

Each of the five focus groups were asked to consider and respond to an identical set of questions, but specifically focusing on their selected topic. A semi-structured focus group schedule was devised by LA, LH and CDR and shared with a senior PA practice partner for further refinement, following an appraisal of Garthwaite et al.’s study, which focused on research-informed advocacy (production, engagement, directions) from health inequalities researchers’ perspectives [44]. Our study expanded on this and involved other key stakeholders, rather than only researchers, to explore similar questions, as related to PA (see Additional file 1). Overarching topics included: focus of future research, what researchers may do beyond regular academic approaches, what a priority research and practice agenda may involve, and facilitators and barriers to developing this agenda. Focus groups were facilitated by members of the Fuse PAN and were audio-recorded. Facilitators encouraged practice partner and researcher perspectives in the discussions.

### Workshop 2: 6th December 2018 (Durham, UK)

This workshop was designed around the imminent publication of updated UK physical activity guidelines. This context was the vehicle for discussing issues of implementing national guidance at a local level. All members of the PAN and wider network were invited. Overall, there were 57 participants (see Table 2), of which 16 had attended workshop one. With the exception of BG, all authors presented or participated in this workshop.

**Table 2:** Workshop 2 participants by employment sector

Participants’ employment sector	***n*** = 57
**Higher education**	**32**
*Academic*	*19*
*Doctoral studies*	*11*
*Professional support*	*2*
**Government**	**19**
*Local authority – health improvement*	*10*
*Local authority – sport development*	*6*
*Civil service*	*2*
*Local authority – service design*	*1*
**National Health Service**	**3**
*Foundation trust management*	*2*
*Physical activity practitioner*	*1*
**Voluntary and commercial**	**2**
*Sport development*	*2*
**National governing bodies (sport)**	**1**

#### Presentations

During the morning, six presentations were given on topics related to findings from workshop one, policy development (including the forthcoming PA guidelines) and implementation. These provided background information and theory to inform later discussions. Speakers included two representatives of the UK’s Department of Health and Social Care and the UK Chief Medical Officers’ Expert Committee for Physical Activity, as well as four academics (three from local universities) and a senior physical activity practitioner.

#### Action planning

Following these presentations, three parallel action planning sessions took place in the afternoon where further data were collected. During these sessions, participants were asked to reflect on the findings of workshop one and the implementation of PA guidelines, to generate ideas for solutions to the identified issues in the local policy implementation context. Participants were asked to identify four key actions, alongside any requirements for research, evidence and knowledge exchange to support these (see Additional file 2). Discussions from each parallel session were fedback to all delegates during a 30-minute plenary session. Action planning sessions, co-ordinated and overseen by CDR, lasted 1 hour and were facilitated by LH, LA and AI who documented sessions through note-taking, production of mind-maps and presentations back to the larger group, which also provided an opportunity to check for accuracy.

### Analyses

#### Workshop one: inductive coding and thematic analysis

Data recordings from workshop one were transcribed verbatim, inputted into QSR NVivo 10 software and analysed by BR (trained in qualitative analysis to postgraduate level) using Thematic Analysis procedures, which emphasised both recurring and important content [45, 46]. We were not able to differentiate speakers (other than facilitator) from the FG recordings; consequently, identification of the individual from which each quotation was elicited was not possible.

Initial complete coding, both data- and researcher-derived, aimed to identify anything relevant to the research aims, particularly the first. This involved assigning labels that reflected semantic and conceptual interpretations of the data [43]. Our research focus on tensions between stakeholder groups meant that coding was inevitably informed by known structural and organisational considerations in physical activity promotion (e.g. financial constraints, co-production or culture shifts), as well as theoretical concepts of interaction, such as whole systems and complexity.

Candidate themes were developed from the data and reflected views and experiences from across all three focus group topics. A senior qualitative researcher (BG) independently coded two transcripts, and a meeting was held between BR and BG for critical discussion and reflection on interpretations by each coder (BR, BG), which in turn facilitated refinement of codes generated by BR. Thereafter, candidate themes were discussed between co-authors at two research meetings (meeting one: BR, BG, LA, LH, CDR; meeting two: BR, CDR, PVDG). Candidate themes from the analysis of workshop one data were presented during workshop two, to inform participant discussions. This also formed a sense-checking mechanism, which fed back into researcher meetings.

#### Workshop two: action plans

Notes and materials produced during workshop two were analysed by CDR through a process of systematically checking for commonly cited terms and overlapping ideas across the parallel groups. These commonalities were summarised in overarching action plans, which were discussed with LA, LH, BR and PVDG to confirm accuracy. Excerpts from the action plans were used as illustrative examples of solutions to the challenges raised in our analyses.

#### Post-workshops

The research process concluded with the development of guiding principles to support PA research, policy and practice. Alongside PVDG (an experienced knowledge exchange broker), BR and CDR discussed and drafted these principles based on the findings from both workshops during a dedicated session. These principles were reviewed by LA and LH, and further revisions were made.

## Results

Three themes, reflecting challenges faced by practice partners and researchers, were identified: i) *understanding complexity and context*; ii) *addressing the knowledge and skills gaps*; and iii) *mismatched timescales and practices*.

### Understanding complexity and context

Workshop participants recognised the need to consider complex processes and different rationalities in their efforts to increase regional PA; *‘It’s not like one size fits all* ***[C2]****’.* However, there was also a perception that these factors are often ill-understood within academia and there is therefore *‘a gap in translating* ***[W1]****’* these ideas into practice.

*Looking at the research […*] *is it individuals, or is it the system that we have to look at, research and understand better how it works? […] Certainly from a practical point of view […] you have to get the entire community to buy into that and buy into the system* ***[I1]***.

*So, how we incorporate that [holistic approach] more […] I don’t think anyone has got their head around [it]. We always then just go back to the “we’re promoting physical activity” type approach. Whereas, I think that’s a really not well understood concept. Probably, a very academic concept, the ‘whole of physical activity’* ***[C1]***.

While the need to account for programmatic complexity (i.e. underlying causes of problems) was acknowledged, participants also stressed the perceived importance of appreciating the unique political and economic complexities in the Northeast region in particular, not least *‘austerity* ***[C2]****.’* It was believed that *‘every community is different, so it’s about understanding what it is they want* ***[I2]****.’* However, the perceived inadequacies of the existing evidence-base were deemed to inhibit stakeholders’ attempts to develop contextually appropriate solutions:

*That’s a big problem, I think […] trying to find out what works in certain places. What works with this type of population and that type of population? [...] It might work where we are, but we don’t know whether it would work there, because maybe our population is just so different ****[C1]***.

Participants acknowledged the need to come together to address these complexities. The following example demonstrates a localised approach in Northumberland:

*We’re going through quite a lot of change […] we’ve got a lot of collaboration and partnership going there […] we’re leading on writing a physical [activity] strategy for Northumberland […] so we’re trying to bring together a bespoke sector - public health, local authority and the NHS - to actually join things up* ***[I2]***.

However, coordinated and collaborative work is complex in itself. This was particularly evident through the way participants articulated their sense of clearly defined stakeholder groups (e.g. researchers, policy-makers or practitioners). There was uncertainty as to *‘what could academics be doing to [better] support practitioners* ***[C1]****’* and likewise, how *‘as a non-academic [one could] approach academics and engage them in research* ***[I1]****.’* Each stakeholder group was perceived to have its own practices, *‘competing agendas* ***[I1]****’* and its own view of what constituted rational engagement with evidence for decision-making. Practice-orientated *‘guidance saying, do this’* being contradicted by ‘*research saying something different* ***[C1]****,’* was considered to be an issue, as one participant explained:

*You read about interventions and you read about the research, and the methods, and everything, and it’s so different to what you have in front of you and your situation and local population, and everything like that* ***[C1]***.

While participants realised the importance of creating *‘links and drawing on certain expertise from those areas to create that productive environment for people to come into [and be active]* ***[I1]****,’* collaborative efforts remained inhibited. Some participants felt that this could be frustrating:

*It is about having those constant relationships and being constantly aware of the things that are coming up […] whenever I go to these meetings, it just seems like, “God, how are we going to get past the difference in the way we do things?* ***[I2]***”

These differences extended to decision-making. Within the context of their own experiences, different participants appeared better able to respond to evidence, in all its forms, and enact particular ways of working. It was felt, particularly among practitioners, that the capacity for rationale evidence-informed decision-making was limited in both the physical activity sector, and the general public. Informational shortcuts, familiarity and norms were important decision-making shortcuts for some participants:

*This many schools are now doing this new [physical activity programme]. I actually think […] that people do think about what everybody else is doing [rather than the academic evidence] [...] everyone drinks coffee, yet, when you look at the scientific literature, physical activity is far more beneficial for raising your attention. But, as practitioners, we still drink coffee. We’re making an irrational decision and we know the evidence-base ****[C1]***.

Furthermore, these distinctions between groups raised unease among participants about their roles and ability to actually come together, while still, each *‘organisation works in silo’* and there is no *‘shared goal* ***[I2]****’* among stakeholders.

Struggles with collaboration seemed strongest among participants who considered themselves to be *‘a bit in the middle’* or *‘with their foot in both camps* ***[C1]****’.* Some felt that they were *‘not really a practitioner* ***[C1]****,’* nor a researcher in the academic sense. That these stakeholders may have an important boundary-spanning role to play did not really feature during discussions. Rather, there was a sense of needing to conform to groups. Overall, the uncertainty around collaboration caused many participants to question whether efforts in the northeast were capturing the ‘bigger picture’ and focus on *‘looking at the right outcomes* ***[I1]****.’* They believed that *‘there’s a lot of learning that we’re missing out on* ***[I1]****.’*

To summarise, focus group participants articulated the need to better address the complexities of PA promotion, which also involves carefully considering the context in which policy programmes and evidence are generated, transferred and implemented. Participants were clear that *‘events like this [Fuse PAN workshops] are very useful* ***[I1]****’* in bringing people together, disrupting established ways of working, coming to terms with others’ views of rational decision-making, and learning to cope with context-specific complexities.

In response to complexity and different rationalities, participants in workshop two suggested developing a systems approach to PA policy implementation as a future action for local- and regional-level PA stakeholders, such as members of the Fuse PAN. Mapping out local services, organisations and policies and considering key partners (e.g. housing and transport) could inform a context-appropriate systems approach and demonstrate how PA might support other local policies (e.g. social engagement). Based on this systems-map, a joint approach could be developed incorporating shared trust, responsibility and transparency across a range of stakeholders.

### Addressing the knowledge and skills gap

The focus group discussions highlighted an overarching theme about sector-wide ‘gaps in skills, knowledge and confidence’ in reducing physical inactivity, which covers myriad concepts beyond the incomplete evidence-base. This theme captured two facets: 1) communication and translation of research and good practice; 2) practitioners and researchers’ education.

The communication and translation of research, best practice and the benefits of PA was considered to be largely substandard. Participants articulated a need to learn how to ‘spread the word’ about PA in a simple and appealing manner.

*About the guidelines [...] it’s really convoluted to pick up and read. It’s 70 pages long for one. I think there is a need for a simple message. So where [presenter] is saying that people have got so much going on in their lives, the context, for them to get their heads around, when their heads are so full of many more important things, we have a simpler message rather than this big convoluted message maybe* ***[I2]***.

Participants felt that across the board professionals and researchers do not communicate their practices and findings in ways that the public or other professionals can understand. They identified a desire to receive these messages in appropriate ways and build on work happening in different sectors.

*Academics or PhD students don’t know how to give information out to non-academics. So, although some of us might have those skillsets, we’re not trained in Design & Technology; we’re not there to create these fun and simplistic ways to feed back to schools* ***[C2].***

While sometimes academics were thought to be poor at translating their research into practice, practitioners were also perceived to be not very good at sharing their good practices:

*In every local authority, [...] there’s a lot of work that happens and no-one hears about it. So, that’s because, maybe, the methods weren’t good enough and you couldn’t publish it, maybe or whatever. I think there’s a lot of learning in there that we’re missing out on* ***[C1].***

Participants wondered how to ‘sell’ science in a culturally salient manner to the general public, as well as key organisations and actors:

*So, if you go into local authorities, you won’t hear anybody advertising, “You should be more physically active.” It’s more like, you know, they’ll use broader terms like “moving more” or whatever. So, the sedentary behaviour messages are, actually, much easier to understand; are we sitting less? So, that’s like, “Do anything, just don’t sit down.” That’s an easier message, isn’t it* ***[C1]****?*

Participants acknowledged that to spread the word professionals and researchers had to become better at demonstrating the value of PA, acknowledging that this is often a hard ‘sell’ with not everybody being willing to change or accept help in reducing inactivity.

*There’s no concrete, “This is what the benefits are that you’ll get for your school.” So, I think it’s a long way off becoming more of an intervention that [is communicated* *via* *stakeholder-relevant messages such as that] exercise is going to improve academic performance or active learning* **[C2]***.*

The ‘selling’ of PA was also considered to be hindered by various competing ideas and initiatives that are being rolled out across the health sector. Better communication, using simple, easy to understand messages about the benefits of PA and translation of research evidence and best practice to other contexts was deemed important.

*I think when it comes to the interventions, I think we have too much of lots of different interventions: so you want to stop smoking, you’ve got to go to the stop smoking services. You want to lose weight, you’ve got to go and see [...] we need more one stop shops, this is about the person, they go to one place and all of their needs get addressed in that place* ***[I2]****.*

A complementary view held by some suggested ‘education is key’ to achieving this; from educational reform in schools to upskilling the workforce, and from enabling practitioners to gain research skills, to academics understanding the landscape in which they intervene. Participants suggested that training for practitioners could look at existing evidence on motivations for people to get involved in physical activities:

*I think whoever is delivering to young people, they need to understand children’s motivations to want to do an activity. So, sometimes, if it’s more of a sports coach, their motivation, themselves, is for the football team to win. The actual children that are attending the session, their motivations are very different as to why they’re attentive. So, I think we definitely need to have some CPD as to the type of person that delivers to those children and whether they’re putting them off activity* ***[C2]****?*

Perhaps more importantly, some participants believed that training should focus on how practitioners can incorporate physical activities in their daily jobs:

*And in Early Years, part of the curriculum is physical development but you don’t have to do PE, if that makes sense? So, physical development means we’ve got a little yard there, with some bikes and a climbing frame and whatever and it’s “Go and help yourself.” If they don’t help themselves, then that’s them done* ***[I1]****.*

It was felt that researchers on the other hand, could benefit from incentive structures that encourage engagement in the practice and implementation landscape:

*There’s this perverse incentive for researchers to finish their research project, publish their paper and move on to the next [and] not [to ask], “How are we now going to make sure that this is as useful as possible for the practitioners?” […] [T]hat’s a challenge* ***[C1]****.*

Participants with a practice background reported a lack of confidence, skills or knowledge in research, which they felt are not addressed adequately in current education programmes for professionals.

*The conflict in the research and the contradictory findings is very confusing sometimes. So ultimately, what you then go into is the review of reviews or a meta-analysis, something like that to just give me the highlights, tell me what, in general, what does work* ***[I1]****?*

Practitioners often felt uncertain about the evidence-base surrounding PA interventions, and that many interventions have differential effects that are currently unknown.

*So, I think, a difficulty, when you read an intervention, is really […] it might work but you don’t know. It might work where we are but we don’t know whether it would [definitely] work there because maybe our population is just so different. Trying to get people to back you [*e.g. *commissioners], with fluffy stuff like that, it’s difficult. Like, if you try and get money from places that are tight on resources, I think that’s very difficult* ***[C1]****.*

Participants thought that research could help to clarify these effects but stressed that professionals need to know how to access, assess and apply the appropriate research evidence. To upskill professionals in research and how to engage with different groups, participants suggested that education change is required but also clarification of roles, which would allow professionals to dedicate time to research and engagement activities. This also requires closer alignment between researchers and providers:

*What we’ve started to do is to work with the organisations who are already working with the people we’re trying to target because what we’re finding is the sports coaches, they don’t have the skills. They’re not equipped to actually go in and deliver effectively. So where people have got mental health issues, we’re working with the providers, the talking therapy service to embed it within the services. So for us it’s not necessarily about targeted programmes but actually it’s about finding the right people to be working with to deliver those programmes* ***[I2]****.*

Participants were keen to learn from other sectors about using and applying research and to understand better their role in the wider system (as individuals, within organisations and communities, and on a regional scale region). More formally, participants suggested that PA should be embedded in medical and allied professions’ higher education pathways and should not been seen as a standalone qualification:

*The GCSE PE came about because they wanted to professionalise PE. So, it got kept and it was just as good as any other subject because it had a qualification attached to it. Now, I think it’s spoiling physical activity levels because who cares if you can dribble a hockey ball? I’d be more caring about whether people are physically active and still doing stuff when they leave school* ***[C2]****.*

In response to the perceived knowledge and skills gap, participants in the second workshop suggested various solutions for adapting policy to context. For example, by tailoring guidelines to local populations or priority groups. Such tailoring would require a more in-depth understanding of what PA means to different groups and how it matches their values and identity. It was suggested that doing so would help to create a context-specific marketing and communication strategy for different local sectors. New national PA guidelines would provide a key opportunity for clear and accessible messaging within local marketing and branding, given an adequate time-frame.

Workshop two participants also acknowledged persistent challenges linked to the complexity of PA promotion, such as a mismatch in time-scales and lack of resources that hinder the implementation of these solutions. Strategies to address these challenges are further discussed within the following theme.

### Mismatched timescales and practices

Participants described a perceived need for a phase shift in working practices across sectors, drawing on the differential timescales for research evidence production (years) versus practice-based strategy and implementation (often much faster) [47, 48]. This tension was apparent in examples provided in discussions:

*[…] because it took us so long, through scoring the observations and training the observers, that when we go back in, to deliver the outcomes and talk about where we go next, they’ve [practice partners] already picked up something else* ***[C1]***.

Further voices ***[I2]*** collectively highlighted that academic time-frames perhaps do not meet societal need for faster-paced practice and implementation. A modern world of fast-paced living was perceived, with PA often associated with constantly evolving technological trends for example ‘*challenges on YouTube’*, ‘*Pokémon GO*’ and ‘*social media*’ more generally ***[C2]***.

There appeared to be agreement that researchers could and should do better in these regards, through ‘*articulating based on their experiences, how effective [a project] may be in other settings* **[C1]**’, that is, providing general insight and finding alternative ways to facilitate moving at scale, rather than rigidly adhering to hierarchies of evidence-generation.

Opportunities for practical solutions and examples of good working practice were discussed in respect of identified constraints and included, for example, accessing a pool of *‘skilled-up students in physical activity* ***[C2]***’ where a faster-paced research approach is required, or where fiscal restraints limit formal research opportunities. More broadly, conversations highlighted potential opportunities to access a ‘*vibrant*’ student body with potential to better-relate to and interact with people ‘*on the ground* ***[C2]***’. There was appetite to overcome a ‘*mismatch between where our priorities are* ***[I1]****’* and build research capacity and skills outside of academia, while increasing researchers’ understanding of local decision-making environments through embedded researchers and studentship opportunities, for example. The strategic role of the Fuse PAN in developing less formalised opportunities was also considered:

*[Fuse PAN could facilitate] interactions about things that are at the point of design*, *or* ‘*surgeries’ […] people may come along and may want some academic input or just a chance to talk through an issue* ***[I1]***.

In summary, researchers’ long-held concerns of not being able to produce publishable findings, or publishing lower quality research were considered problematic when working with shorter timeframes for applied practice initiatives. Times were perceived to have changed to be quicker-paced, dynamic and technologically driven. There was recognition that traditional research approaches may not always be fit for purpose or map onto the practices or pace of the current ‘real-world’ PA context.

In response to the expressed need of participants to align practices more closely, workshop two participants suggested that a key action plan for the Fuse PAN going forward would be to change policy priorities at the local level, with a need to move PA up the agenda locally and secure more resources to support this agenda. Funding was perceived to be lacking for key activities identified earlier in response to the second theme (addressing the knowledge and skills gap), such as marketing, communication, collaboration and associated training and upskilling. While participants recognised the challenge of securing more resources in times of austerity and public sector cuts, they suggested behavioural and cultural changes that require limited or no additional resources, such as recognition of achievements and lived experiences of ‘experts in the field’, such as refugee communities and children’s groups (e.g. via participatory action research and invitations to forums, such as future Fuse PAN workshops). Participants agreed that there was a need to enable better working practices between stakeholders, facilitate better understanding of local programmes, and support the adaptation of national policies to local context. They suggested this might be achieved via time-efficient evaluation methods.

In sum, the findings from across both workshops have provided insight into how Fuse PAN members perceived the complexities of promoting PA in local contexts. However, it was thought that through embracing this complexity, rather than resisting it, opportunities for progress may be found. PA research, policy and practice may be supported by creating diverse and inclusive spaces for discussion and evidence exchange, with an emphasis on simple and focused messaging, which can generate timely, practical and easily implemented solutions to the issues that stakeholders face.

## Discussion

### Principal findings

In this paper we presented a case study of a cross-sectoral network in Northeast England (Fuse PAN) to identify local-level challenges and opportunities for implementing PA interventions more broadly, and in particular the recently updated UK PA guidelines. Our findings demonstrate that implementing national PA policy in local practice and decision-making is a complex process. This presents researchers, policy-makers and practitioners with several challenges that require effective collaboration in order to generate system-level improvements [13, 30]. There is therefore a need for specific skills, particularly in communicating and translating research evidence in continuously changing local contexts. Furthermore, it may necessitate a broader culture shift, toward a more integrated approach to research alongside practice in participatory and action-oriented approaches, which redefines stakeholders’ roles.

Our findings highlight the need to develop new skills and ways of working and reinforce calls for a whole systems approach to implementation of policy and research [49, 50]. This approach necessitates: i) embracing complexity in implementing national policies locally; ii) recognising that PA policies are connected to, and impact on, many other local policies; iii) adapting policies and associated communication strategies for different local groups across sectors (e.g. [51]) to help bridge the gap between stakeholders’ perceptions of the value of research; and iv) advocating PA as a local priority for funding to support other policy objectives. Developments of this nature are ongoing, most notably through Sport England’s local delivery pilots [52].

While complementing the emerging knowledge-base, the views and experiences of stakeholders also emphasise the enduring nature of the challenges associated with, and possible reforms necessary to facilitate, whole system PA promotion. A particularly stubborn challenge is the mismatch between the time taken to conduct research and the immediacy with which evidence is needed in policy and practice [53]. Policy-makers and practitioners often need to make quick decisions in rapidly changing environments, yet evidence on which to inform decisions about how best to increase PA engagement is not always readily available. Our findings revealed that stakeholders believe that ensuring PA becomes a well-resourced local priority will not speed up the research process. This and many other challenges identified by stakeholders have persistently been raised in the PA literature [1, 54, 55]. This will likely continue despite progress in translational research, a problem which is indicative of the wicked nature of these issues and the need to understand and manage their complexity.

Our study extends the knowledge-base by providing a local and practical perspective on stimulating a whole system approach amid the competing policies and priorities of various stakeholder groups, who discussed difficulties in attaining and evaluating holistic intervention. While the importance of policy was recognised, there is need to explore a systems approach built on structures and policies, together with tailor-made programmes to suit specific contexts in which people live. Understanding these local contexts, and the people who operate within and across them, will help both implementation and utilisation [56, 57].

Our findings also revealed that developing clear communication strategies are thought to be vital. Communicating research in different and relatable ways to different audiences is an integral part of translational research, yet this skill is often overlooked [57]. Upskilling professionals in research and how to engage different groups will require university curricula and professional development courses to better reflect insights from translational research and systems thinking [58]. Additionally, the roles of researchers and practitioners need to be clarified and dedicated collaborative research and sense-making should be incentivised.

### Lessons from Fuse PAN for adapting national PA policy to local context

Local networks of policy-makers, practitioners and researchers, such as Fuse PAN, provide a practical interface to overcome incompatible individual behaviours of policy-makers, practitioners and researchers, as previously suggested [30]. Collaborative research events, like the Fuse PAN workshops, provide a platform for researchers, practitioners and policy-makers to collectively explore the complexity of local decision-making systems, and explore mechanisms to deal with it. These workshops are also a valuable opportunity to disrupt ways of working and confront different rationalities. They enable open-minded conversation about the use and value of different types of evidence (or the lack of it) and how to communicate it more effectively to different stakeholder groups, as well as integrate it in shared decision-making processes.

Collaborative research events are important steps in developing responsive and flexible systems approaches to implementing PA policy [1, 13, 59]; however alone, they are insufficient. As the Fuse knowledge exchange model indicates, there are possibly four steps to translating evidence into policy and practice, which are closely linked and interactive [34]. Collaborative research events, such as the Fuse PAN workshops, contribute to the first two steps: 1) awareness raising (i.e. network mailing list; website as a resource for dissemination; and social media coverage); and 2) knowledge sharing (i.e. workshops with a mix of academics (students, early, mid and senior academics) and policy and practice partners, which operates as a platform for discussing and developing research, and changing policy and practice). However, as highlighted by this study, further progress may be made by making evidence fit for purpose (step 3) and supporting the uptake and implementation of PA research evidence in local systems (step 4).

To enable this and to consolidate the awareness raising (step 1) and knowledge sharing (steps 2) of the knowledge exchange model, communication needs to be tailored to different audiences, by producing research and practice briefs, and disseminating research such as this paper, which synthesise and reflect on stakeholders’ insights. Previous research has demonstrated the need for clear communication between actors at different levels of the system across policy, practice and research [30]. Our study provides examples of what this communication could look like at the local level.

To progress into step 3 of the model, it is proposed that local networks of policy-makers, practitioners and researchers consider creating research development groups [60] consisting of interested parties to advance local ideas in funding proposals in a route for development, evaluation and future implementation. This step would address significant challenges for PA policy implementation highlighted in previous studies (i.e. inadequate capacity, inappropriate outcome measures, and insufficient funds [26].

Furthermore, local networks could respond to enquiries from policy and practice partners, and co-create knowledge with partners, which will help to ensure that evidence is fit for the local context.

In our experience of promoting PA, there is space to create further embedded roles across research, policy and practice (e.g. professors of practice, or PhD candidates), in which stakeholders from different sectors can spend time elsewhere (i.e. staff secondment). This will help build research capacity and skills outside of academia, while increasing researchers’ understanding of local decision-making environments.

Longer-term, these activities may lead local networks of policy makers, practitioners and researchers into step 4: supporting uptake and implementation of evidence. Successful knowledge exchange will depend on effectively linking activities across each step. Local networks can be a driver for capacity building and collaborative PA research across the region and elsewhere. Workshops and other events, such as those discussed here, can be the catalyst for focused discussion between commissioners, funders and research development groups, who can action and resource generated ideas. This will ultimately help to achieve convergence of top-down and bottom-up approaches [13, 23] by successfully facilitating locally implementation of nationally designed PA policy, and creating a feedback loop to inform future national policy-making.

### Strengths and limitations

The qualitative research design, with inclusion of policy-makers, practitioners and academics together with solution-focused workshops, add depth and understanding to the issues raised in the literature and Fuse PAN case study. The focus group facilitators were mostly academic researchers and based in Fuse, which may have affected the responses of participants. To ensure that our findings captured the breath of views in the PAN, we also held the second workshop, which offered opportunities for in-depth discussion of the issues and allowed practitioners and researchers to be challenged. Our primary interest was to collate views from stakeholders across different physical activity sectors, and as such this is what our results reflect. We acknowledge, as a limitation, that we are unable to refer to specific individuals for analysis.

Our findings are based on a localised case study in Northeast England, with its particular history of development, and a unique policy and practice context. Nevertheless, the challenges and opportunities for implementation are applicable to other UK regions, or globally. Furthermore, the findings do not represent an inscrutable nor definitive approach to addressing PA. Despite these limitations, our analysis of the focus group data reached thematic saturation and our findings are in line with previous studies [48, 61–63], which suggests a degree of trustworthiness of the results. Moreover, while different issues may apply in other countries with different governance and health systems, the literature suggests the ubiquity of these concerns [64]. Challenges in translating national PA policy and guidance to local levels are experienced across the globe and will continue to drive a need to bring together policy-makers, practitioners and researchers for addressing the complexity of PA promotion.

## Conclusions

Our case study of the Fuse PAN highlights persistent barriers for translating national PA policy and guidelines into policy and practice. Local policy-makers, practitioners and academic researchers articulated the need to better address the complexities of PA promotion. Networks, such as Fuse PAN, are useful for bringing people together, disrupting ways of working, coming to terms with different rationalities, and learning to cope with a region’s complexities. Times were perceived to have changed and there was recognition that traditional research approaches may not always be fit for purpose or map onto the practices or pace of the current ‘real-world’ PA context.

To support this way of working new skills are required of each profession, particularly in communicating and translating research in continuously changing local contexts. This may necessitate a broader culture shift, toward a more integrated approach to research alongside practice in participatory and action-oriented approaches, which redefines PA stakeholders’ roles. These findings reinforce calls for a whole systems approach to implementation of policy and research.

The updated UK PA guidelines policy presents an opportunity for multiple agencies to consider context-specific implementation and to address enduring tensions experienced across stakeholders. An organically derived localised implementation plan would consider prioritisation of PA, mapping links across other relevant local policies and developing a communication strategy which is relevant to local context.

To guide the local implementation of national PA policy and allow others with an interest in localised PA promotion to benefit from our experiences over the last 7 years, we propose 10 guiding principles that may support knowledge exchange activities and be transferred to other contexts:

### Guiding principles for implementing national PA policy at a local level

- Embrace the complexity of implementing national policies.
- Recognise that different rationalities about evidence-use exist across sectors and places.
- Create space to bring different rationalities together, challenge the status quo and debate the value and use of national policy and evidence.
- Accept different perspectives and think outside of the box to create multidisciplinary spaces (e.g. sport and exercise sciences, public health or social sciences).
- Be pragmatic: follow the energy and ideas created by networks, as well as recent national developments (e.g. a Fuse PAN workshop focused on the relative merits of using digital to support PA).
- Do not be deterred by limited resources – harness organic development.
- Frame messages in simple, easily accessible language that is tailored for different stakeholder groups.
- Foster inclusive leadership: every member can play their part and have a voice in decision-making.
- Have a clear vision and look beyond targeted interventions to wider systems approaches and determinants of health.
- Ensure structural engagement between partners beyond formal meetings to sustain timely and pro-active knowledge exchange activities.

## Supplementary Information

**Additional file 1.** Topic list for focus groups (workshop 1).

**Additional file 2.** Discussion topics for workshop 2.

## Data Availability

The datasets used and/or analysed during the current study (e.g. anonymised focus group transcripts and description of coding tree) are available from the corresponding author on reasonable request.

## Abbreviations

Fuse PAN: Fuse’s Physical Activity Network
PA: Physical activity
UK: United Kingdom

## Footnotes

1 Fuse PAN workshops are biannual and, on average, attract 80 delegates from different sectors, including policy, practice and academia.
