# Supplementary material for: Challenges, opportunities and solutions for local physical activity stakeholders: an implementation case study from a cross-sectoral physical activity network in Northeast England
Source: BMC Public Health. 2020 Nov 23;20:1760. doi: 10.1186/s12889-020-09847-3 (PMC7686773; doi:10.1186/s12889-020-09847-3)
Supplement: Supplementary file 1 — Additional file 1. Topic list for focus groups (workshop 1). [file 12889_2020_9847_MOESM1_ESM.pdf]

Topic list for focus groups (workshop 1)

1. What are the gaps in the evidence-based around supporting populations or individuals to be physically active in the topic area your workshop is focusing on (i. physical activity inequalities; ii physical activity in children and young people; or iii. physical activity in the workplace)? We are particularly interested in understanding what practitioners need to know.

2. What is important for researchers to be doing beyond academic work to support efforts to reduce physical activity inequalities; increase physical activity in children and young people; or increase physical activity in the workplace?

3. What are the priority areas for physical activity-related research in each topic area?

4. What will facilitate this research to be conducted and how can barriers for translation into practice be overcome?
