# Supplementary material for: Challenges, opportunities and solutions for local physical activity stakeholders: an implementation case study from a cross-sectoral physical activity network in Northeast England
Source: BMC Public Health. 2020 Nov 23;20:1760. doi: 10.1186/s12889-020-09847-3 (PMC7686773; doi:10.1186/s12889-020-09847-3)
Supplement: Supplementary file 2 — Additional file 2. Discussion topics for workshop 2. [file 12889_2020_9847_MOESM2_ESM.pdf]

Discussion topics for workshop 2

Guidance was provided to delegates via a slide (presented by TK) that asked them specifically to consider ‘*What does the northeast of England need to do to successfully implement the new PA guidelines?*’.

The slide also provided three discussion topics (developed by TK, CDR, and BR):

1. *What activities will you be responsible for in the implementation of the new PA Guidelines?*

2. *Will the new PA Guidelines require you to change how you work – e.g. undertake new tasks or new ways of working, or work with new groups?*

3. *What challenges need to be overcome to implement the new PA Guidelines successfully, and are these new challenges or continuing ones?*
